# Trabecular bone structural parameters evaluated using dental cone-beam computed tomography: cellular synthetic bones

**DOI:** 10.1186/1475-925X-12-115

**Published:** 2013-11-09

**Authors:** Jung-Ting Ho, Jay Wu, Heng-Li Huang, Michael YC Chen, Lih-Jyh Fuh, Jui-Ting Hsu

**Affiliations:** 1 School of Dentistry, College of Medicine, China Medical University, Taichung 404, Taiwan; 2 Department of Biomedical Imaging and Radiological Science, China Medical University, Taichung 404, Taiwan; 3 Department of Dentistry, China Medical University and Hospital, Taichung 404, Taiwan

## Abstract

**Objective:**

This study compared the adequacy of dental cone beam computed tomography (CBCT) and micro computed tomography (micro-CT) in evaluating the structural parameters of trabecular bones.

**Methods:**

The cellular synthetic bones in 4 density groups (Groups 1–4: 0.12, 0.16, 0.20, and 0.32 g/cm^3^) were used in this study. Each group comprised 8 experimental specimens that were approximately 1 cm^3^. Dental CBCT and micro-CT scans were conducted on each specimen to obtain independent measurements of the following 4 trabecular bone structural parameters: bone volume fraction (BV/TV), specific bone surface (BS/BV), trabecular thickness (Tb.Th.), and trabecular separation (Tb.Sp.). Wilcoxon signed ranks tests were used to compare the measurement variations between the dental CBCT and micro-CT scans. A Spearman analysis was conducted to calculate the correlation coefficients (*r*) of the dental CBCT and micro-CT measurements.

**Results and Conclusion:**

Of the 4 groups, the BV/TV and Tb.Th. measured using dental CBCT were larger compared with those measured using micro-CT. By contrast, the BS/BV measured using dental CBCT was significantly less compared with those measured using micro-CT. Furthermore, in the low-density groups (Groups 1 and 2), the Tb.Sp. measured using dental CBCT was smaller compared with those measured using micro-CT. However, the Tb.Sp. measured using dental CBCT was slightly larger in the high-density groups (Groups 3 and 4) than it was in the low density groups. The correlation coefficients between the BV/TV, BS/BV, Tb.Th., and Tb.Sp. values measured using dental CBCT and micro-CT were 0.9296 (*p* < .001), 0.8061 (*p* < .001), 0.9390 (*p* < .001), and 0.9583 (*p* < .001), respectively. Although the dental CBCT and micro-CT approaches exhibited high correlations, the absolute values of BV/TV, BS/BV, Tb.Th., Tb.Sp. differed significantly between these measurements. Additional studies must be conducted to evaluate using dental CBCT in clinical practice.

## Introduction

The trabecular bone is a sponge like bone composed of numerous trabeculae. Previous studies have reported that trabecular bone thicknesses ranges from 200 and 400 μm and the structure varies depending on the bone function and location in the body [1-4]. Several structural parameters are used to represent the architecture of the trabecular bone, namely, the bone volume fraction (BV/TV), trabecular number (Tb.N.), trabecular thickness (Tb.Th.), and trabecular separation (Tb.Sp.) [5]. In the past, histology methods were typically employed to assess trabecular bone structure. However, histological methods were invasive and therefore inappropriate for use in clinical diagnoses. In addition, histological methods provided only limited 2D information, which is inadequate for representing comprehensive 3D structures.

Computed tomography (CT) is used to stack 2D X-ray slices to reconstruct a 3D image and is the current mainstream method for assessing trabecular bone structure. In general, micro computed tomography (micro-CT) and high-resolution peripheral quantitative computed tomography (HR-pQCT) have been used for evaluating trabecular bone structures. Micro-CT produces image slices at a resolution ranging from 7 and 35 μm and is typically used for analyzing the trabecular bone structures of small animals and small human biopsy specimens [6,7]. Previous studies have confirmed highly obtained highly accurate analyses by using micro-CT and referencing histological methods [8]. Micro-CT is typically considered the standard for evaluating trabecular bone structure. However, because of scanning range restrictions and excessive radiation doses, micro-CT is limited to small animal models [7] and human biopsy specimen analyses [6,8], and is unsuitable for clinical diagnoses. Regarding clinical and in vivo studies, HR-pQCT has become increasingly prevalent because display resolution has increasingly enhanced. HR-pQCT produces slices at resolutions ranging from 70 to 300 μm [9,10] and is typically used for analyzing the peripheral trabecular bone structure of human distal long bones. In previous studies [3,4], researchers have employed HR-pQCT to analyze human distal radii and distal tibiae, determining the relationships between the structural parameters and mechanical properties of trabecular bones. However, the scanning range of HR-pQCT is tailored to measuring the periphery of distal long bones; this method is inadequate for analyzing craniofacial bones.

Recently, dental cone-beam computed tomography (CBCT) has become increasingly prevalent among dental clinics. Compared with conventional CT, dental CBCT presents numerous advantages such as lower radiation doses, shorter acquisition times, superior resolution, and affordable cost [11]. Because of technological advancements, the resolution of current dental CBCT ranges from 80 to 400 μm [12,13], which is similar to that of HR-pQCT. However, most previous studies have used only a single grayscale value in the dental CBCT to represent the host bone quality, and have not evaluated the structural parameters of the trabecular bone [11,14-16]. In our previous studies [17-21], these structural parameters, and particularly the bone to implant contact percentage (BIC %), have significantly influenced the dental implant’s primary stability, which is the key factor in its survival rate [6,18,22]. Therefore, elucidating the structure of the trabecular bone prior to dental implant surgery would be invaluable.

Previous studies have employed micro-CT for evaluating trabecular bone parameters; however, few studies have examined using dental CBCT to predict the structural parameters of the trabecular bone [23,24]. Therefore, we compared the adequacy of dental CBCT and micro-CT for evaluating the structural parameters of trabecular bones.

## Materials and methods

### Specimen preparation

**Figure 1:**
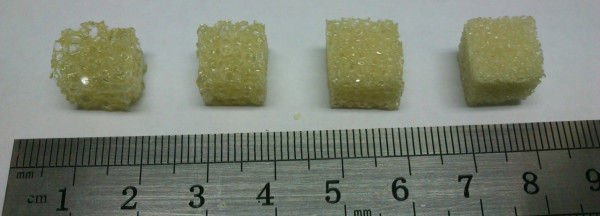
**The cellular synthetic bone used in this study [from left (Group 1) to right (Group 4): 0.12 g/cm**^**3**^**, 0.16 g/cm**^**3**^**, 0.20 g/cm**^**3**^**, and 0.32 g/cm**^**3**^**].**

Cellular synthetic bones were used in this study because obtaining fresh human cadaver bones is difficult. Four rigid and synthetic cellular polyurethane bones (Sawbones, Vashon, WA, USA) representing trabecular bones with densities of 0.12 g/cm^3^ (Model 1522–09, Group 1), 0.16 g/cm^3^ (Model 1522–10, Group 2), 0.20 g/cm^3^ (Model 1522–11, Group 3), and 0.32 g/cm^3^ (Model 1522–12, Group 4) were used in this study (Figure 1). Each specimen (dimensions of 1 cm^3^) was prepared to simulate the structure of peri-dental implant bone. The range of the density and elastic moduli of the trabecular bones used herein were based on those of Misch et al. [25]. Each group consisted of eight specimens. Overall, 32 cellular synthetic bone specimens were examined using dental CBCT and micro-CT scans.

### Dental CBCT and micro-CT scanning and trabecular bone parameter measurements

The specimens were scanned in air to reduce the interference of surrounding materials in the CBCT and micro-CT analyses. A dental CBCT device (AZ 3000, Asahi Roentgen, Japan) was used to generate dental CBCT images of each specimen. The scanning parameters were set to 85 kV, 3 mA, a rotation step of 0.7°, and a voxel resolution of 100 μm. The dental CBCT scanning images were archived using the DICOM file format and 16-bit grayscale units. Micro-CT images of each specimen were obtained using the SkyScan 1076 micro-CT (SkyScan, Aartselaar, Belgium). The scanning parameters were set to 48 kV, 149 uA, a rotation step of 0.4°, and a voxel resolution of 17.24 μm. The scanned raw micro-CT images were archived using the TIF file format and 16-bit grayscale units. After scanning, the raw micro-CT images were imported into an imaging reconstruction software program, NRecon 1.6.9 (SkyScan, Aartselaar, Belgium). During the reconstruction step, the level of beam hardening correction was set at 100% to reduce inhomogeneous grayscale representation in the micro-CT images.

Regarding the micro-CT and dental CBCT approaches, the images were imported to ImageJ 1.46r (Rasband, W.S., ImageJ, U.S. National Institutes of Health, Bethesda, MD, USA). The BoneJ 1.3.9 program [26] was applied to measure the 4 trabecular bone structural parameters: BV/TV, BS/BV, Tb.Th., and Tb.Sp. (Table 1). Before analyzing the trabecular bone parameters, both the CBCT and micro-CT images processed filtering, thresholding, and binarizing steps. In filtering step, contrast and despeckle filters were applied. In the thresholding step, the “optimized threshold” method of the BoneJ program was applied to determine the threshold that yielded minimal connectivity levels. The voxel-counting method was applied to analyze the bone volume and total volume. Regarding the bone surface analysis, triangular surface mesh (2 pixels of side length) was applied. During the Tb.Th. and Tb.Sp. analyses, the diameter of the largest sphere that fit within the structure or hollow space was measured.

**Table 1:** Trabecular bone structure parameters measured in this study

**Indices**	**Abbrev.**	**Unit**	**Definition**
Bone volume fraction	BV/TV	%	Ratio of the segmented bone volume to the total volume
Specific bone surface	BS/BV	mm^-1^	Ratio of the segmented bone surface to the segmented bone volume
Trabecular thickness	Tb.Th.	mm	Mean thickness of the trabeculae
Trabecular separation	Tb.Sp.	mm	Mean distance between the trabeculae

### Statistical analysis

The mean, standard deviation, and coefficient of variation (CV) were calculated for all measurements. Wilcoxon signed ranks tests were used to compare the variations between the dental CBCT and micro-CT measurements. A Spearman analysis was conducted to calculate the correlation coefficients (*r* values) between the dental CBCT and micro-CT measurements. All statistical analyses were performed using OriginPro software (Version 8, OriginLab, Northampton, MA, USA). The statistical significance level was set to *p* < .05.

## Results

**Figure 2:**
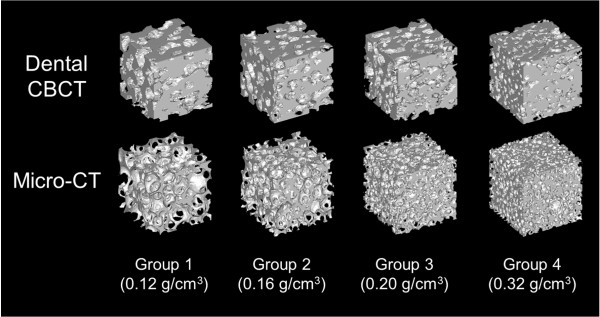
The 3D models of the cellular synthetic bone created by using dental CBCT and micro-CT.

Figure 2 shows 3D models of the cellular synthetic bone specimens which compiled using micro-CT and dental CBCT. In addition, Table 2 presents the 4 trabecular bone structural parameters that were calculated using these approaches. In Groups 1–4, both the BV/TV measured using the dental CBCT and micro-CT approaches and the BS/BV measured using the dental CBCT approach increased regarding enhanced specimen density. By contrast, the Tb.Th. and Tb.Sp. decreased as the specimen density was enhanced. Regarding the BS/BV measured using micro-CT, the BS/BV of Group 3 (density: 0.20 g/cm^3^) was slightly larger than was that of Group 4 (density: 0.32 g/cm^3^). However, the slight variation was statistically non-significant. In the 4 groups, the BV/TV and Tb.Th. measurements obtained using dental CBCT were all larger than were those obtained using micro-CT (*p* < .001). By contrast, the BS/BV measurements obtained using dental CBCT were significantly lower than were those obtained using micro-CT (*p* < .001). Furthermore, in the low-density groups (1 and 2), the Tb.Sp. measurements obtained using dental CBCT were lower than were those obtained using micro-CT. By contrast, the Tb.Sp. measurements obtained using dental CBCT were slightly larger in the high-density groups (3 and 4) compared with the low-density groups. Moreover, in all groups, the coefficient of variation of the BV/TV, BS/BV, and Tb.Th. measurements obtained using dental CBCT were higher compared with those obtained using micro-CT.

**Table 2:** The trabecular bone structural parameters measured by using dental CBCT and micro-CT

**Group**		**1**	**2**	**3**	**4**
Density		0.12 g/cm^3^	0.16 g/cm^3^	0.20 g/cm^3^	0.32 g/cm^3^
	Parameter	Mean	Mean	Mean	Mean
	(unit)	±SD (CV%)	±SD (CV%)	±SD (CV%)	±SD (CV%)
dental CBCT	BV/TV	0.3813	0.4171	0.4495	0.5041
	(%)	±0.0365 (9.58)	±0.0298 (7.15)	±0.0344 (7.65)	±0.0346 (6.86)
	BS/BV	3.8478	4.3609	4.5422	5.4714
	(mm^-1^)	±0.1903 (4.95)	±0.2119 (4.86)	±0.2842 (6.26)	±0.3903 (7.13)
	Tb.Th.	1.0155	0.8774	0.8249	0.6434
	(mm)	±0.0514 (5.06)	±0.0390 (4.44)	±0.0375 (4.55)	±0.0313 (4.86)
	Tb.Sp.	1.8209	1.3698	1.1385	0.7184
	(mm)	±0.0947 (5.20)	±0.0356 (2.60)	±0.0312 (2.74)	±0.0288 (4.01)
micro-CT	BV/TV	0.1603	0.1949	0.2589	0.3590
	(%)	±0.0151 (9.42)	±0.0065 (3.34)	±0.0122 (4.72)	±0.0056 (1.56)
	BS/BV	9.4327	10.4456	11.6841	11.4896
	(mm^-1^)	±0.4077 (4.32)	±0.2628 (2.52)	±0.3627 (3.10)	±0.1631 (1.42)
	Tb.Th.	0.4169	0.3734	0.3280	0.3123
	(mm)	±0.0160 (3.83)	±0.0117 (3.14)	±0.0078 (2.37)	±0.0037 (1.18)
	Tb.Sp.	2.2985	1.7038	1.0578	0.7064
	(mm)	±0.2143 (9.32)	±0.0920 (5.40)	±0.0392 (3.71)	±0.0532 (7.53)

*SD*, Standard deviation; *CV*, Coefficient of variation (SD/Mean); *BV/TV*, Bone volume fraction; *BS/BV*, Specific bone surface; *Tb.Th*., Trabecular thickness; *Tb.Sp*., Trabecular separation.

**Figure 3:**
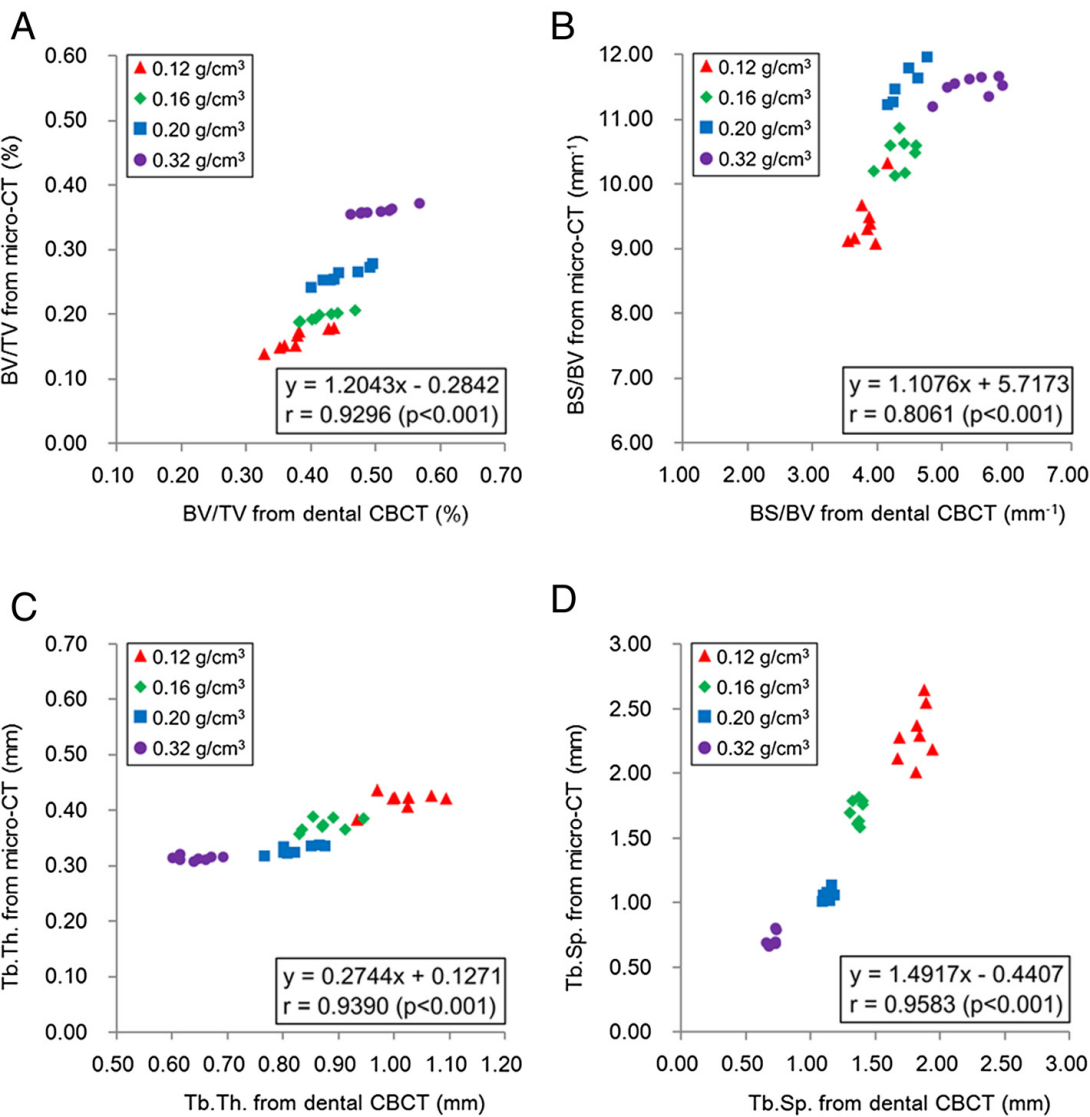
**Correlations of trabecular bone structure parameters measured using dental CBCT and micro-CT. (A)** BV/TV; **(B)** BS/BV; **(C)** Tb.Th., and **(D)** Tb.Sp.

Figure 3 shows the correlations between the dental CBCT and micro-CT measurements. The correlation coefficients between the BV/TV, Tb.Th., and Tb.Sp. measurement values obtained using dental CBCT and micro-CT were 0.9296 (*p* < .001), 0.9390 (*p* < .001), and 0.9583 (*p* < .001), respectively, exhibiting a high correlation. The correlation between the BS/BV measurement value obtained using dental CBCT and micro-CT was 0.8061 (*p* < .001). In addition, the correlations between the 4 BV/TV and the other 3 trabecular bone structural parameters (BS/BV, Tb.Th., and Tb.Sp.) measured using micro-CT (*r* = 0.7485, -0.8959, -0.9641 for BS/BV, Tb.Th., and Tb.Sp., respectively, *p* < .001) were all stronger than were those of the BV/TV parameters measured using dental CBCT (*r* = 0.5307, -0.5963, -0.9210 for BS/BV, Tb.Th., and Tb.Sp., respectively, *p* < .001).

## Discussion

Typically, the peri-implant trabecular bone and cortical bone severely affect the dental implant’s primary stability [17-21], which is a critical factor influencing its survival rate [6,18,22]. Among the structural parameters of trabecular bones, BIC% has been confirmed to directly influence this primary stability [17,19]. Therefore, it is crucial to understand the trabecular bone structures of the jawbone before inserting a dental implant. Histological analyses have been the most directly method of assessing trabecular structure [8,27]; however, it is invasive and failed to provide 3D structural information. Although micro-CT has been adopted as the standard for analyzing trabecular bone structures, it is unsuitable for use in clinical diagnoses because of scanning range restrictions. Recently, the resolution of dental CBCT has improved, achieving resolutions similar to those yielded by HR-pQCT (80–400 μm). Nevertheless, most studies adopting dental CBCT have regarded the trabecular bone as a non-porous homogeneous structure, using a single grayscale value or bone mineral density for assessment. Thus, this study is the first to assess the structural parameters of cellular synthetic trabecular bones by using dental CBCT and micro-CT, comparing the results yielded by both systems. The findings indicated that although the absolute values differed, the results of both methods were highly correlated. These results may serve as a reference for clinical practitioners who employ dental CBCT to analyze the trabecular bone structures of alveolar bones.

Dental CBCT has been widely used in clinical dental practice; however, because of the restrictions caused by insufficient resolution, most dentists assess bone quality by examining the average CBCT grayscale values within or around the implant placement area before conducting dental implant surgery [14,16,22]. One study indicated a moderate correlation between implant survival rate and CBCT bone quality analysis [22]. In addition, González-García et al. applied micro-CT to perform bone biopsies following dental implantation, comparing the results to the presurgical CBCT analysis [14]; the findings showed a high correlation between the BV/TV analyses of micro-CT images and the grayscale analyses of CBCT images [14]. However, only moderate to low levels of correlation were determined between other trabecular bone structural parameters (e.g., Tb.Th., Tb.Sp.) after comparing the micro-CT and grayscale CBCT images.

In the current study, the BV/TV and Tb.Th. values measured using CBCT were higher than were those measured using micro-CT. Ibrahim et al. attained similar results [24] after examining human cadaver specimens. In addition, in the high-density specimens (Groups 3 and 4), yielded slightly higher Tb.Sp. measurements using dental CBCT compared with using micro-CT. However, the low-density specimens (Groups 1 and 2) yielded lower Tb.Sp. using dental CBCT compared with using micro-CT, contradicting the results of Ibrahim et al.’s study [24]. This implied that distinct porosity levels among specimens might yield varied structural analyses when comparing the results of dental CBCT and micro-CT. Regarding structural complexity, a previous study reported that dental CBCT yielded lower trabecular numbers compared with micro-CT [24]. In the current study, we used BS/BV as the structural complexity index, obtaining similar results as a previous study [24]. In the current study, the Tb.Th. measured using micro-CT ranged from 0.307 to 0.435 mm and the mean was 0.358 mm for cellular synthetic bone specimens; this was similar to the Tb.Th. of human jawbones reported in previous studies [1,2,6]. The Tb.Th. measured using CBCT approximately twice to that measured using micro-CT. This discrepancy could result from partial volume effects [28]. Another reason was the large voxel size required for CBCT scanning; thin trabeculae might not be detected, causing increased Tb.Th. and decreased BS/BV values in the CBCT images. Although though the experimental results were distinct, the structural parameters were highly correlated between the CBCT and micro-CT image analyses.

Previous research of trabecular bone structures has primarily examined natural bone and cellular synthetic bone specimens and each bone demonstrated unique advantages [29]. Natural bones are organic biochemical structures that are inhomogeneous, anisotropic, and viscoelastic, whereas cellular synthetic bones are easily obtainable, and the density and porosity of these specimens can be controlled. Because obtaining fresh human cadaver bone was difficult, cellular synthetic bone specimens composed of polyurethane were used in this study. In previous studies, various sources were used to obtain varying-sized specimens of maxillary bone to analyze the structures of trabecular bones. In clinical practice, dental implantation surgeons could harvest cylinder bone biopsies approximately 5 mm in diameter by using trephine before inserting dental implants [6,14]. In laboratory practice, entire dry cadaver mandibles or sections of dentition regions have been prepared as experimental specimens [23]. In this study, cubic sawbones specimens (length = 1cm) were applied to simulate the bone structures of peri-dental implants; these structures have been reported to significantly affect implant survival rates [6,18,22].

Threshold methods have been thoroughly discussed for use in micro-CT systems [30,31], however, discussions regarding thresholding CBCT images have remained scarce. Naitoh et al. proposed a dental CBCT image threshold method based on the varying proportions of cortical bone and water grayscale values [23], suggesting that the BV/TV measurements would exhibit significant variations as these proportions changed. This study involved an optimized threshold method built in the BoneJ program. This method has been proven to demonstrate similar analysis accuracy levels as other software programs such as the Scanco (Brüttisellen, Switzerland) and SkyScan CTAn (Aartselaar, Belgium) devices [26]. The threshold method in this study used all pixels in a stack to construct a histogram, employing the built-in isodata algorithm of ImageJ to determine the threshold. The threshold that yielded minimal connectivity was subsequently employed in the binarization step. In addition, because this method was sensitive to image noise, all images were masked using the contrast and despeckle filters before determining their thresholds. A previous study reported CBCT images that exhibited lower signal to noise ratios compared with micro-CT images [32]. In the current study, filtering also increased the signal to noise ratio. An alternate study indicated that an increased signal to noise ratio could compensate for the disadvantages caused by a large image voxel size [33].

Certain limitations to this study should be mentioned. First, the cellular synthetic bone specimens used in this study were not prepared using bone mineral material. Thus, the results cannot indicate the ability of dental CBCT systems to analyze human cadaver bones. Second, during clinical dental CBCT scanning, the quality of CBCT images is affected by surrounding tissues. However, these effects were not examined in this study and all specimens were scanned in air. Third, the same analysis software, BoneJ analysis software, was applied to the dental CBCT and micro-CT images. However, this software cannot measure the trabecular number, which is regarded as the basic trabecular bone structural parameter. Finally, this study involved analyzing only cellular synthetic bone specimens that comprised 4 distinct densities. The adequacy of dental CBCT in analyzing the trabecular bone structure of specimens that exhibit other densities requires further exploration.

## Conclusion

Dental CBCT and micro-CT systems were to assess 4 trabecular bone structural parameters (BV/TV, BS/BV, Tb.Th., and Tb.Sp.) of 4 cellular synthetic bone specimens of varying densities. The absolute values of the experimental results obtained using dental CBCT significantly differed from those obtained using micro-CT. However, the results yielded by the two instruments demonstrated a strong positive correlation. However, additional studies are necessary to validate the use of dental CBCT in clinical studies.

## Competing interests

The authors have no competing interests to declare.

## Authors’ contributions

JTH, HLH, and JW conceived and designed the experiments; MYCC, JTH, and LJF performed the experiments; HLH and JTH analyzed the data; JTH wrote the manuscript; and all authors read and approved the final version of manuscript.

## References

[B1] Fanuscu MI Chang T-L Three-dimensional morphometric analysis of human cadaver bone: microstructural data from maxilla and mandible Clin Oral Implants Res 2004 15 2 213 218 10.1111/j.1600-0501.2004.00969.x 15008933

[B2] Moon H-S Won Y-Y Kim K-D Ruprecht A Kim H-J Kook H-K Chung M-K The three-dimensional microstructure of the trabecular bone in the mandible Surg Radiol Anat 2004 26 6 466 473 10.1007/s00276-004-0247-x 15146293

[B3] Liu XS Zhang XH Sekhon KK Adams MF McMahon DJ Bilezikian JP Shane E Guo XE High-resolution peripheral quantitative computed tomography can assess microstructural and mechanical properties of human distal tibial bone J Bone Miner Res 2010 25 4 746 756 19775199 10.1359/jbmr.090822 PMC3130204

[B4] Boutroy S Rietbergen BV Sornay-Rendu E Munoz F Bouxsein ML Delmas PD Finite element analysis based on in vivo HR-pQCT images of the distal radius is associated with wrist fracture in postmenopausal women J Bone Miner Res 2008 23 3 392 399 17997712 10.1359/jbmr.071108

[B5] Bouxsein ML Boyd SK Christiansen BA Guldberg RE Jepsen KJ ller RM Guidelines for assessment of bone microstructure in rodents using micro-computed tomography J Bone Miner Res 2010 25 7 1468 1486 10.1002/jbmr.141 20533309

[B6] Huang H-L Hsu J-T Chen MYC Liu C Chang C-H Li Y-F Chen K-T Microcomputed tomography analysis of particular autogenous bone graft in sinus augmentation at 5 months: differences on bone mineral density and 3D trabecular structure Clin Oral Investig 2013 17 2 535 542 10.1007/s00784-012-0725-1 22526892

[B7] Bagi CM Berryman E Moalli MR Comparative bone anatomy of commonly used laboratory animals: implications for drug discovery Comp Med 2011 61 1 76 85 21819685 PMC3060425

[B8] González-García R Monje F Is micro-computed tomography reliable to determine the microstructure of the maxillary alveolar bone? Clin Oral Implants Res 2013 24 7 730 737 10.1111/j.1600-0501.2012.02478.x 22540518

[B9] Bagi CM Hanson N Andresen C Pero R Lariviere R Turner CH Laib A The use of micro-CT to evaluate cortical bone geometry and strength in nude rats: correlation with mechanical testing, pQCT and DXA Bone 2006 38 1 136 144 10.1016/j.bone.2005.07.028 16301011

[B10] Genant HK Engelke K Prevrhal S Advanced CT bone imaging in osteoporosis Rheumatology 2008 47 Suppl 4 iv9 iv16 18556648 10.1093/rheumatology/ken180 PMC2427166

[B11] Mah P Reeves T McDavid W Deriving Hounsfield units using grey levels in cone beam computed tomography Dentomaxillofacial Radiol 2010 39 6 323 335 10.1259/dmfr/19603304 PMC3520236 20729181

[B12] Patel S New dimensions in endodontic imaging: Part 2. Cone beam computed tomography Int Endod J 2009 42 6 463 475 10.1111/j.1365-2591.2008.01531.x 19298576

[B13] Vos WD Casselman J Swennen GRJ Cone-beam computerized tomography (CBCT) imaging of the oral and maxillofacial region: a systematic review of the literature Int J Oral Maxillofac Surg 2009 38 6 609 625 10.1016/j.ijom.2009.02.028 19464146

[B14] González-García R Monje F The reliability of cone-beam computed tomography to assess bone density at dental implant recipient sites: a histomorphometric analysis by micro-CT Clin Oral Implants Res 2013 24 8 871 879 10.1111/j.1600-0501.2011.02390.x 22250839

[B15] Isoda K Ayukawa Y Tsukiyama Y Sogo M Matsushita Y Koyano K Relationship between the bone density estimated by cone-beam computed tomography and the primary stability of dental implants Clin Oral Implants Res 2012 23 7 832 836 10.1111/j.1600-0501.2011.02203.x 21545533

[B16] Salimov F Tatli U Kürkçü M Akoğlan M Öztunç H Kurtoğlu C Evaluation of relationship between preoperative bone density values derived from cone beam computed tomography and implant stability parameters: a clinical study Clin Oral Implants Res 2013 doi: 10.1111/clr.12219. [Epub ahead of print] 10.1111/clr.12219 23772811

[B17] Hsu J-T Huang H-L Chang C-H Tsai M-T Hung W-C Fuh L-J Relationship of Three-Dimensional Bone-to-Implant Contact to Primary Implant Stability and Peri-implant Bone Strain in Immediate Loading: Microcomputed Tomographic and In Vitro Analyses Int J Oral Maxillofac Implants 2013 28 2 367 374 10.11607/jomi.2407 23527336

[B18] Huang H-L Chang Y-Y Lin D-J Li Y-F Chen K-T Hsu J-T Initial stability and bone strain evaluation of the immediately loaded dental implant: an in vitro model study Clin Oral Implants Res 2011 22 7 691 698 10.1111/j.1600-0501.2010.01983.x 21054551

[B19] Hsu J-T Huang H-L Tsai M-T Wu AY-J Tu M-G Fuh L-J Effects of the 3D bone-to-implant contact and bone stiffness on the initial stability of a dental implant: micro-CT and resonance frequency analyses Int J Oral Maxillofac Surg 2013 42 2 276 280 10.1016/j.ijom.2012.07.002 22867739

[B20] Huang H-L Tu M-G Fuh L-J Chen Y-C Wu C-L Chen S-I Hsu J-T Effects of elasticity and structure of trabecular bone on the primary stability of dental implants J Med Biol Engin 2010 30 2 85 89

[B21] Hsu J-T Fuh L-J Tu M-G Li Y-F K-TC MS Huang H-L The effects of cortical bone thickness and trabecular bone strength on noninvasive measures of the implant primary stability using synthetic bone models Clin Implant Dent Relat Res 2013 15 2 251 261 10.1111/j.1708-8208.2011.00349.x 21599830

[B22] Molly L Bone density and primary stability in implant therapy Clin Oral Implants Res 2006 17 S2 124 135 10.1111/j.1600-0501.2006.01356.x 16968388

[B23] Naitoh M Aimiya H Hirukawa A Ariji E Morphometric analysis of mandibular trabecular bone using cone beam computed tomography: an in vitro study Int J Oral Maxillofac Implants 2010 25 6 1093 1098 21197484

[B24] Ibrahim N Parsa A Hassan B Stelt P Aartman IHA Wismeijer D Accuracy of trabecular bone microstructural measurement at planned dental implant sites using cone-beam CT datasets Clin Oral Implants Res 2013 doi: 10.1111/clr.12163. [Epub ahead of print] 10.1111/clr.12163 23581278

[B25] Misch CE Qu Z Bidez MW Mechanical properties of trabecular bone in the human mandible: implications for dental implant treatment planning and surgical placement J Oral Maxillofac Surg 1999 57 6 700 706 10.1016/S0278-2391(99)90437-8 10368096

[B26] Doube M Kłosowski MM Arganda-Carreras I Cordelières FP Dougherty RP Jackson JS Schmid B Hutchinson JR Shefelbine SJ BoneJ: Free and extensible bone image analysis in ImageJ Bone 2010 47 6 1076 1079 10.1016/j.bone.2010.08.023 20817052 PMC3193171

[B27] Schmitt CM Doering H Schmidt T Lutz R Neukam FW Schlegel KA Histological results after maxillary sinus augmentation with Straumann® BoneCeramic, Bio-Oss®, Puros®, and autologous bone. A randomized controlled clinical trial Clin Oral Implants Res 2013 24 5 576 585 10.1111/j.1600-0501.2012.02431.x 22324456

[B28] Adams JE Quantitative computed tomography Eur J Radiol 2009 71 3 415 424 10.1016/j.ejrad.2009.04.074 19682815

[B29] Poukalova M Yakacki CM Guldberg RE Lin A Saing M Gillogly SD Gall K Pullout strength of suture anchors: effect of mechanical properties of trabecular bone J Biomech 2010 43 6 1138 1145 10.1016/j.jbiomech.2009.12.007 20117785 PMC2849904

[B30] Hara T Tanck E Homminga J Huiskes R The influence of microcomputed tomography threshold variations on the assessment of structural and mechanical trabecular bone properties Bone 2002 31 1 107 109 10.1016/S8756-3282(02)00782-2 12110421

[B31] Waarsing JH Day JS Weinans H An improved segmentation method for in vivo microCT imaging J Bone Miner Res 2004 19 10 1640 1650 10.1359/JBMR.040705 15355559

[B32] Schulze R Heil U Groβ D Bruellmann D Dranischnikow E Schwanecke U Schoemer E Artefacts in CBCT: a review Dentomaxillofacial Radiol 2011 40 5 265 273 10.1259/dmfr/30642039 PMC3520262 21697151

[B33] Bechara B McMahan CA Moore WS Noujeim M Geha H Teixeira FB Contrast-to-noise ratio difference in small field of view cone beam computed tomography machines J Oral Sci 2012 54 3 227 232 10.2334/josnusd.54.227 23047033

